# Bank vole prion protein extends the use of RT-QuIC assays to detect prions in a range of inherited prion diseases

**DOI:** 10.1038/s41598-021-84527-9

**Published:** 2021-03-04

**Authors:** Tze How Mok, Akin Nihat, Connie Luk, Danielle Sequeira, Mark Batchelor, Simon Mead, John Collinge, Graham S. Jackson

**Affiliations:** MRC Prion Unit at UCL, Institute of Prion Diseases, Courtauld Building, 33 Cleveland Street, London, W1W 7FF UK

**Keywords:** Prion diseases, Prions

## Abstract

The cerebrospinal fluid (CSF) real-time quaking-induced conversion assay (RT-QuIC) is an ultrasensitive prion amyloid seeding assay for diagnosis of sporadic Creutzfeldt–Jakob disease (CJD) but several prion strains remain unexplored or resistant to conversion with commonly used recombinant prion protein (rPrP) substrates. Here, bank vole (BV) rPrP was used to study seeding by a wide range of archived post-mortem human CSF samples from cases of sporadic, acquired and various inherited prion diseases in high throughput 384-well format. BV rPrP substrate yielded positive reactions in 70/79 cases of sporadic CJD [Sensitivity 88.6% (95% CI 79.5–94.7%)], 1/2 variant CJD samples, and 9/20 samples from various inherited prion diseases; 5/57 non-prion disease control CSFs had positive reactions, yielding an overall specificity of 91.2% (95% CI 80.1–97.1%). Despite limitations of using post-mortem samples and our results’ discrepancy with other studies, we demonstrated for the first time that BV rPrP is susceptible to conversion by human CSF samples containing certain prion strains not previously responsive in conventional rPrPs, thus justifying further optimisation for wider diagnostic and prognostic use.

## Introduction

Prion disease represents a heterogeneous group of rare, transmissible and fatal neurodegenerative conditions affecting humans and other mammalian species, unified biologically by autocatalytic templated-misfolding of the host-encoded prion protein (PrP) into disease-associated forms^1^. In spite of this singular mechanism of disease, the human conditions exhibit striking phenotypic diversity even within sporadic Creutzfeldt–Jakob disease (CJD), which comprises 80–85% of cases. Aside from the classical triad of rapid cognitive decline, gait ataxia and/or myoclonus, distinct clinical phenotypes such as the Heidenhain variant, ataxic variant (Brownell–Oppenheimer), corticobasal syndrome and sporadic fatal insomnia have been observed^2^. This diversity is hypothesised to be imparted by prion strains through the propagation in the brain of different pathological conformational states^1–3^. There is evidence that this process is modified considerably by the polymorphic codon 129 of the prion protein gene (*PRNP*). Strains can be inferred by laboratory assays including by western blot^1,4,5^. Highly-penetrant disease-causing *PRNP* mutations cause diseases historically classified into Gerstmann-Straussler-Scheinker (GSS) syndrome, fatal familial insomnia (FFI) and familial CJD (fCJD)^6^, although this fails to encapsulate the diversity of phenotypes resulting from octapeptide repeat insertions (OPRIs) and the more recently described peripheral systemic amyloidosis secondary to the *PRNP* Y163X mutation^7,8^.

Cerebrospinal fluid (CSF) examination has long been a key clinical investigation in individuals suspected to have sporadic CJD, in particular the detection of protein 14.3.3, raised S100B and exceedingly high total tau levels (typically > 1000 pg/mL) are all considered to be highly supportive^9–11^. Though highly sensitive, the above are not seen exclusively in prion disease, and moreover are really surrogate markers of rapid axonal injury and gliosis; as such, histological examination endures in epidemiological case definitions as mandatory for definite diagnosis^12^.

More recently, real-time quaking-induced conversion (RT-QuIC) seeded by CSF has emerged as a rapid, sensitive (> 90%) and highly specific tool (≈100%) for ante mortem diagnosis in sCJD^13,14^. The RT-QuIC exploits and accelerates the seeded amyloidosis of PrP through repeated cycles of mechanical agitation, coupled with Thioflavin T (ThT) fluorescence read-outs that can be monitored in real time. The typical RT-QuIC reaction mixture, composed vitally of a buffer, recombinant PrP (rPrP) and ThT, has been successfully seeded by a variety of mammalian tissue and bodily fluids^15–18^. The efficiency and sensitivity of the reaction can be further enhanced by altering conditions such as raising temperature, pH and addition of sodium dodecyl sulphate (SDS)^19^, or by using different rPrP constructs, giving rise to the so-called second generation IQ-CSF RT-QuIC^20,21^.

The extraordinary success of RT-QuIC in clinical diagnosis is at present restricted to sCJD and sCJD-like IPD phenotypes, mainly *PRNP* E200K. Save for a single study in CSF samples from patients with P102L and D178N mutations which successfully seeded RT-QuIC reactions using human rPrP^22^, the level of sensitivity has not been replicated in reactions seeded by CSF from GSS-causing IPD mutations (P102L, A117V, etc.) and variant CJD (vCJD) even with IQ-CSF RT-QuIC above. Other IPD mutations are simply too rarely encountered such that CSF may not be available for testing. Crucially, the lack of PrP sequence homology is expected to present a significant barrier to conversion, based on previous P102L and A117V human PrP transgenic mouse modelling experiments^23,24^. Thus, the expectation that a single rPrP type can serve as a ‘universal acceptor’ seemed improbable until some experiments showed efficient transmission of multiple prion strains in bank voles and in transgenic mice expressing bank vole rPrP (BV rPrP)^25,26^, indicating a lack of both strain and species barriers. BV rPrP as a 'universal acceptor' gained further traction when a series of RT-QuIC experiments seeded by brain homogenate (BH) not only demonstrated seeding activity in variant CJD and GSS cases due to a number of *PRNP* mutations which were hitherto undetectable, but also in other mammalian species^15^.

Here, we have explored the universality of BV rPrP as the substrate in RT-QuIC reactions seeded by CSF from a variety of human prion diseases and attempt to adapt the RT-QuIC technique to a 384-well microplate in place of the conventional 96-well format for higher throughput. The wealth of longitudinal clinical data gathered from the UK National Prion Monitoring Cohort (NPMC) since 2008 allowed us to then determine whether the RT-QuIC seeding dose in post-mortem CSF correlates with the rate of functional decline in patients with sCJD^27,28^.

## Results

### Patients

A total of 101 archived post-mortem (PM) CSF samples from autopsy-proven prion disease cases were identified; this included sCJD (n = 79), vCJD (n = 2) and IPD (n = 20) cases. The range of *PRNP* mutations in the IPD group encompassed P102L (n = 5), A117V (n = 2), Y163X (n = 1), D178N (n = 2), E196K (n = 1), E200K (n = 3), 4-OPRI (n = 2) and 6-OPRI (n = 4). Control PM CSF samples were obtained from 49 anonymised autopsy-proven non-prion disease cases. An further set of 8 non-prion PM CSF samples were identified from the NPMC cohort from patients suspected to have sCJD clinically in life but PM examination revealed other causes of illnesses and deaths; PM CSF control samples amounted to 57 in total. A summary of patient demographics, including the *PRNP* codon 129 genotype where applicable and molecular strain type^5^ is available in Table 1. The PM intervals (number of days between dates of death and post mortem examination) are available for all but 3 of our prion disease cases. The mean PM interval is 5.7 ± 3.4 days; that for the anonymised control cases is not known.

**Table 1:** Summary of patient demographics, *PRNP* codon 129 and molecular strain type where available.

	sCJD	vCJD
Mean AAO (years)	64	36
Mean duration (months)	9.4	14.25
Male	35	2
Female	42	0
RT-QuIC positive	70	1 (MV)
RT-QuIC negative	9	1 (MM)
Codon 129MM	36	1
Codon 129MV	21	1
Codon 129VV	13	0
Codon 129 unknown	9	0
**Molecular strain type (London classification)**		
1	0	
2	20	
3	16	
4	0	2
Not detected	1	
Not available	42	
**Clinical syndrome**		
Classical	26	
Ataxic	15	
Cognitive	21	
Psychiatric	6	
Visual	5	
Stroke-like	0	
Sleep/thalamic	2	
Corticobasal syndrome	1	
Not available	3	

This table summarises the relevant demographics from sporadic (sCJD) and variant CJD (vCJD) patients from whom CSF samples were obtained at post mortem which were included in the RT-QuIC assays. This covers mean age at onset (AAO), duration of illness in months, gender, RT-QuIC results, *PRNP* codon 129 genotype (some untested), molecular strain type (London Classification^48^) and clinical syndrome.

### Optimisation of bank vole rPrP RT-QuIC assay

**Figure 1:**
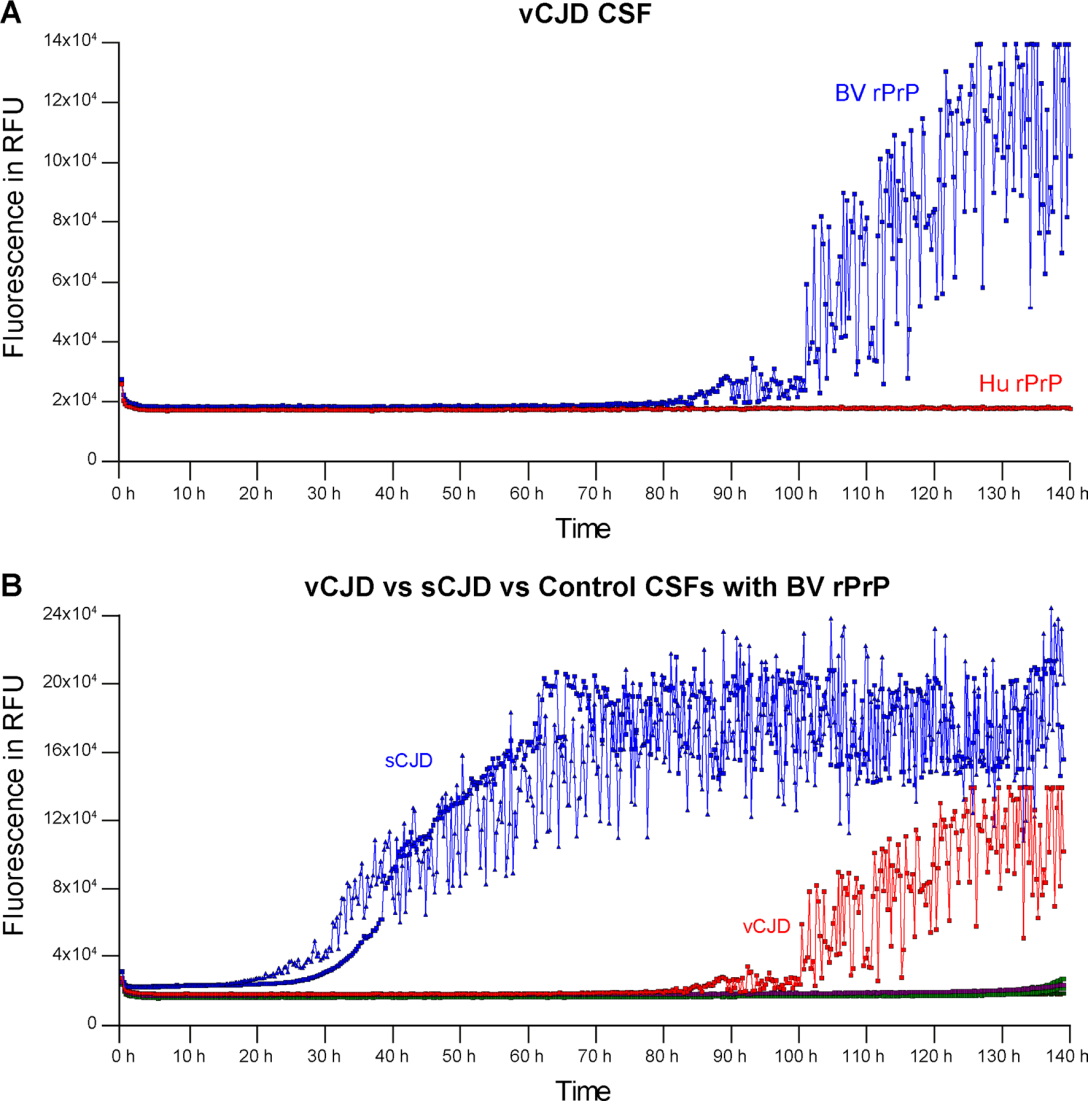
Comparisons between RT-QuIC reactions seeded by CSF samples from sCJD and vCJD cases, using either Hu rPrP or BV rPrP. Each curve is the mean with standard deviation of 4 replicate wells. (**A**) RT-QuIC reactions using BV rPrP (blue) and Hu rPrP (red) in 96-well format at 45 °C, seeded by vCJD CSF; BV rPrP showed a positive result (2/4) but with a long lag phase while Hu rPrP was negative (0/4). (**B**) Comparison of RT-QuIC reactions using BV rPrP, seeded by CSFs from sCJD (blue) and vCJD (red) cases at 45 °C; reactions seeded by sCJD CSFs showed the classic positive curves while that seeded by vCJD CSF replicated the delayed positive curve in (**A**).

First, we compared the RT-QuIC assays using human (Hu) rPrP and BV rPrP as substrates in the standard 96-well plate format at 45 °C, seeded by sCJD (n = 13) and vCJD (n = 1) CSFs against control CSFs. Under the same conditions, RT-QuIC reactions using BV rPrP were positive for all sCJD CSFs (13/13), as opposed to 12/13 for reactions using Hu rPrP. Furthermore, the RT-QuIC reaction with BV rPrP was successfully seeded by vCJD but not using Hu rPrP, albeit with a longer lag phase and lower maximal relative fluorescence units (rfu) in the former compared to sCJD (Fig. 1).

Following that, RT-QuIC experiments were conducted with BV rPrP seeded by sCJD and vCJD cases against control CSFs in both final volumes of 50 μL (CSF 7.5 μL) and 75 μL (CSF 11.25 μL) per well respectively in 384-well plates to see if the reaction kinetics are replicated in smaller well volumes. Both final well volumes of 50 μL and 75 μL produced consistently positive reactions for both sCJD and vCJD, but at lower maximal rfu.

**Figure 2:**
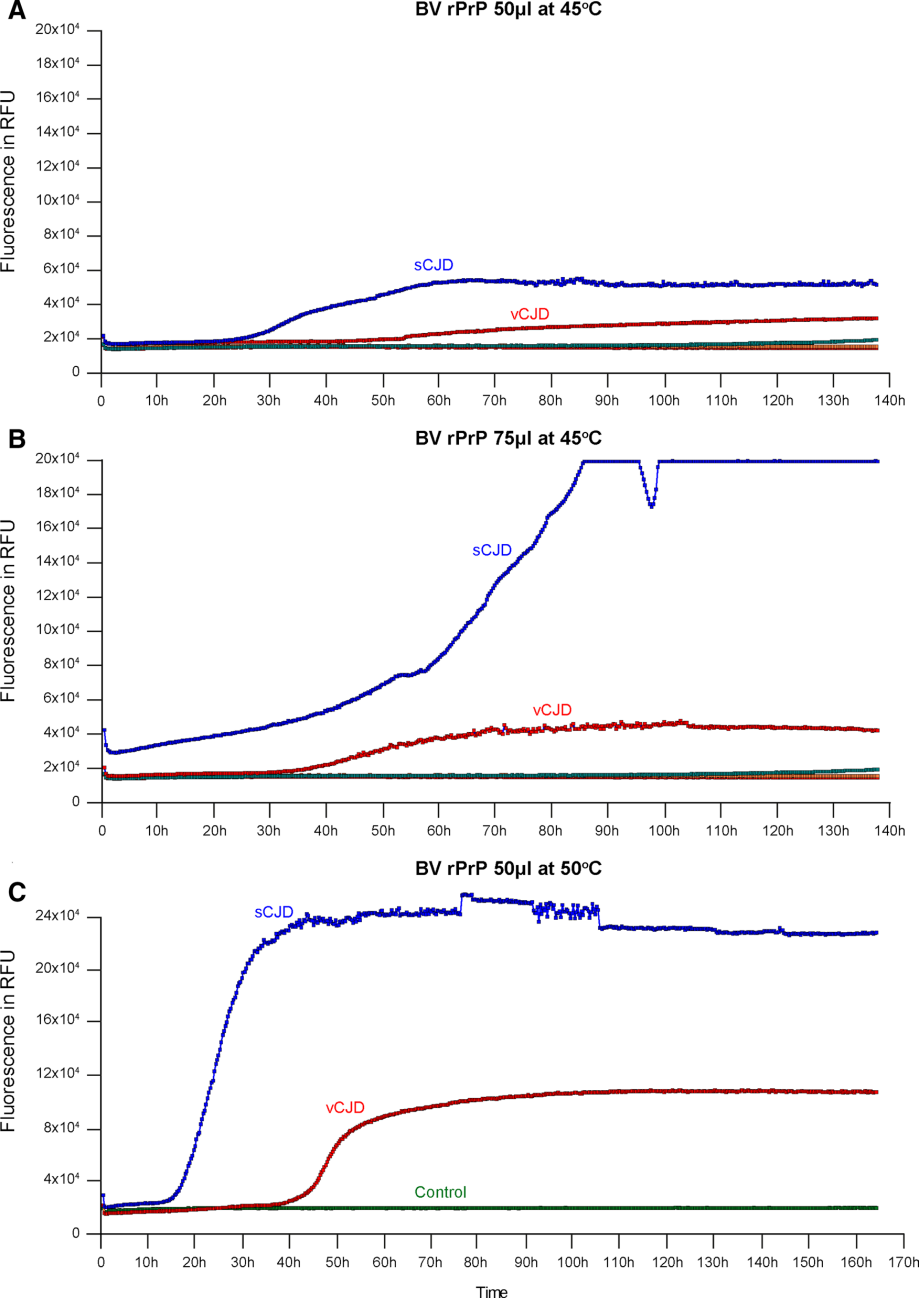
Comparisons of reaction volumes in RT-QuIC reactions using BV rPrP in 384-well format. Reactions seeded by CSF samples from sCJD and vCJD at (**A**) total volume of 50  μL at 45 °C, (**B**) total volume of 75 μL at 45 °C and (**C**) total volume of 50 μL at 50 °C. Each curve is the mean with standard deviation of 4 replicate wells. This series of experiments showed that positive results can be replicated at lower reaction and analyte volumes; raising the temperature to 50 °C improved the efficiency of the reaction by reducing the lag phase and by producing a higher maximal rfu.

Finally, we then performed BV RT-QuIC reactions with 50 μL final volumes at 50 °C in 384-well plates. This showed enhanced maximal rfu for the positive reactions in wells seeded by sCJD and vCJD cases, replicating reaction kinetics seen in those conducted at final volumes of 100 μL in 96-well plates at 45 °C (Fig. 2).

### Bank vole rPrP RT-QuIC reactions seeded by PM CSFs

**Figure 3:**
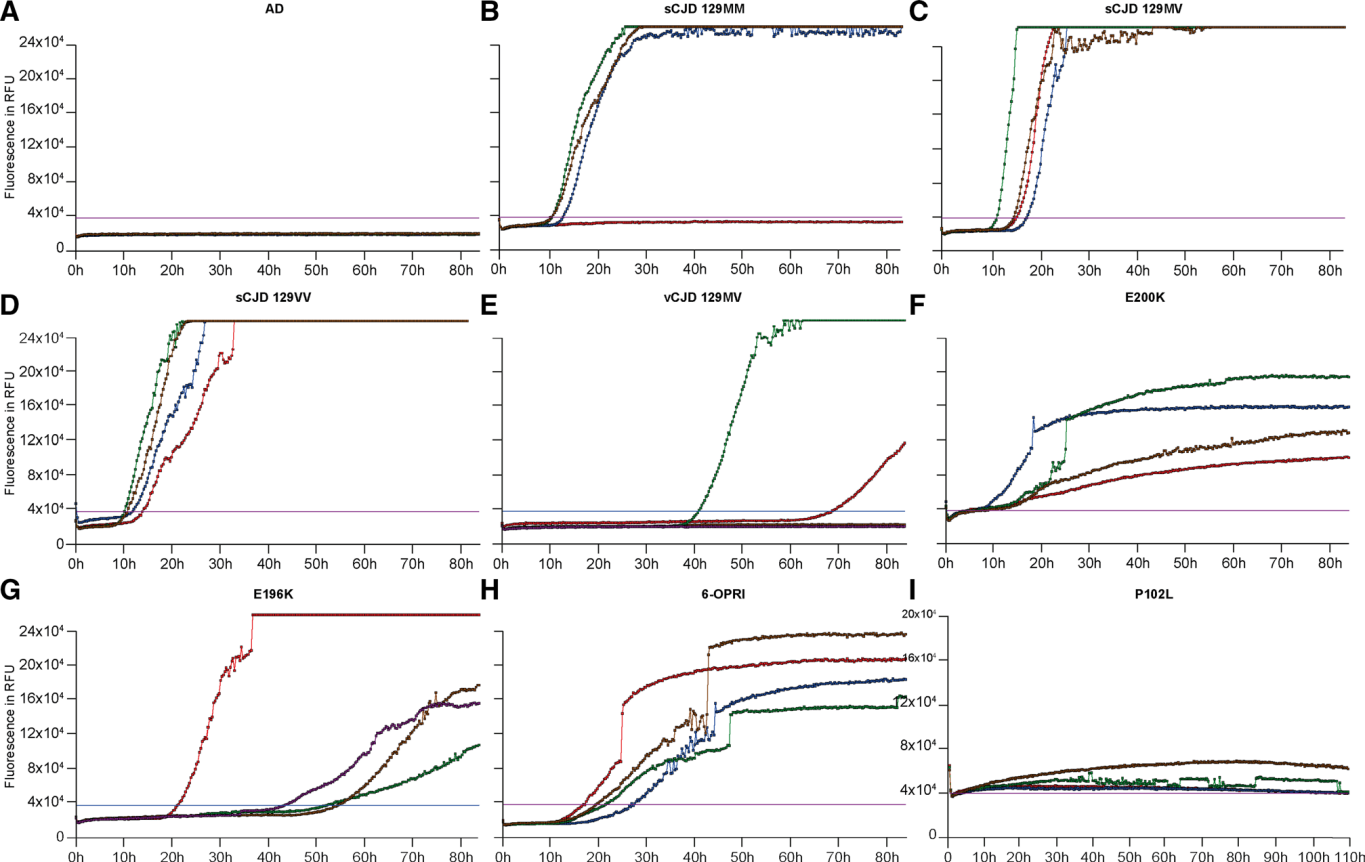
Sample traces of RT-QuIC reactions using BV rPrP in 384-well format. Reaction volumes of 50 μL at 50 °C seeded by CSF from (**A**) Alzheimer’s Disease (AD), (**B**) sCJD 129MM, (**C**) sCJD 129MV, (**D**) sCJD 129VV, (**E**) vCJD 129MV, (**F**) E200K, (**G**) E196K, (**H**) 6-OPRI and (**I**) P102L. Positive results were seen in sCJD with different *PRNP* codon 129 genotypes, vCJD and a range of IPD mutations, while the reaction seeded by AD CSF remained negative throughout. Individual coloured signal curves in each panel represent the signal curves of each well (of which there are 4) of the corresponding prion species/type indicated.

Once optimal conditions were determined (50 μL final well volume (7.5 μL CSF) at 50 °C), BV RT-QuIC reactions were run in 384-well plates seeded by prion disease PM CSFs and non-prion non-neurological disease control PM CSFs (Fig. 3). In 79 cases of definite sCJD, 70 CSF samples were scored positive while 9 were negative; 5 out of 57 non-prion control PM CSF samples were scored positive solely based on exceeding the rfu threshold. This gave a sensitivity of 88.6% (95% CI 79.5–94.7%) and specificity of 91.2% (95% CI 80.7–97.1%) for sCJD cases.

In the other subtypes, BV RT QuIC was positive in 1 of 2 vCJD, and 9/20 IPD [3 of 3 E200K, 1 of 5 P102L, 1 of 1 E196K, 1 of 2 D178N, 0 of 2 A117V, 0 of 1 Y163X, 0 of 2 4-OPRI and 3 of 4 6-OPRI cases (Table 2)].

**Table 2:** Post mortem CSF RT-QuIC results according to types of prion disease (including individual disease-causing *PRNP* mutations), and non-prion disease controls.

	RT-QuIC positive	RT-QuIC negative	Sensitivity %	Specificity%
sCJD	70	9	88.6	
vCJD	1	1	50	
P102L	1	4	20	
A117V	0	2	0	
Y163X	0	1	0	
D178N	1	1	50	
E196K	1	0	100	
E200K	3	3	100	
4-OPRI	0	2	0	
6-OPRI	3	1	75	
All *PRNP* mutations			45	
Non-prion disease controls	5	52		91.2

This table shows that breakdown of CSF RT-QuIC results according to type of prion disease including sporadic CJD (sCJD), variant CJD (vCJD) and individuals *PRNP* mutations (P102L, A117V, Y163X, D178N, E196K, E200K, 4-OPRI, 6-OPRI). The sensitivity and specificity of bank vole rPrP RT-QuIC for sCJD is 88.6% (95% CI 79.5–94.7%) and 91.2% (95% CI 80.7–97.1%) respectively, as opposed to the overall sensitivity of all *PRNP* mutations of 45%.

Application of the Fisher’s Exact Test to compare RT-QuIC results between sCJD and non-sCJD prion disease groups resulted in *p* = 0.001*.*

### Correlation of rate of decline in MRC scale score with prion seeding dose

**Figure 4:**
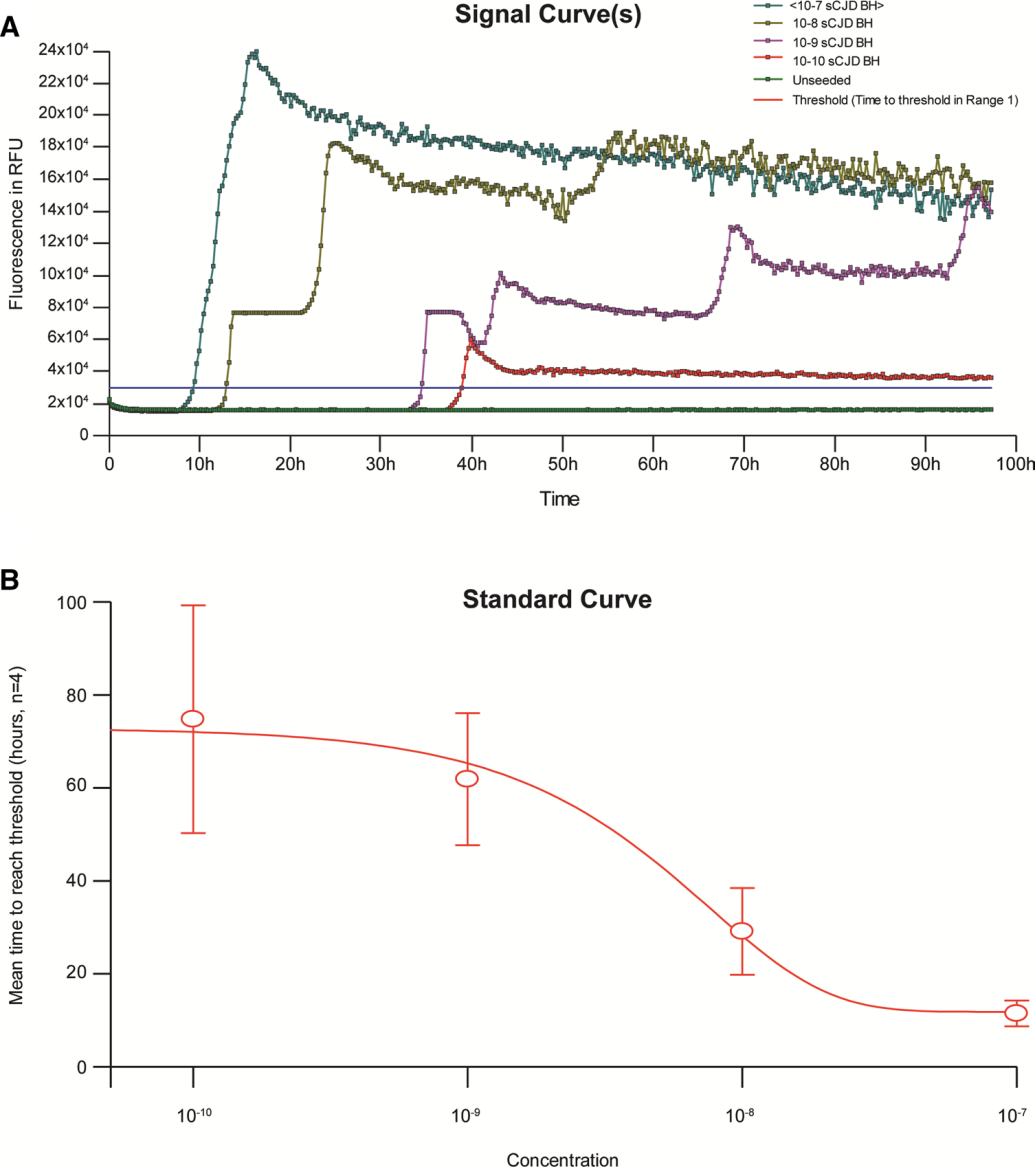
Estimation of CSF prion seeding doses. RT-QuIC reactions seeded by serial dilutions of sCJD 129MM brain homogenates at 50 °C ; each curve representing the average rfu readings across replicate wells (**A**), and dose response curve for estimation of prion seeding doses based on time to threshold lag phases (**B**). Estimation of CSF prion seeding doses were derived from the standard calibration curve (**B**) constructed from the lag phases (time to threshold rfu) of RT-QuIC reactions with BV rPrP seeded by serial dilutions of sCJD BH in (**A**). Data points in (**B**) represent the mean of 4 wells with standard errors of the mean.

Similar to Shi et al*.*^29^, estimation of CSF prion seeding doses were derived from a standard calibration curve constructed from the lag phases (time to threshold rfu) of BV rPrP RT-QuIC reactions seeded by serial dilutions of sCJD BH (Fig. 4).

**Figure 5:**
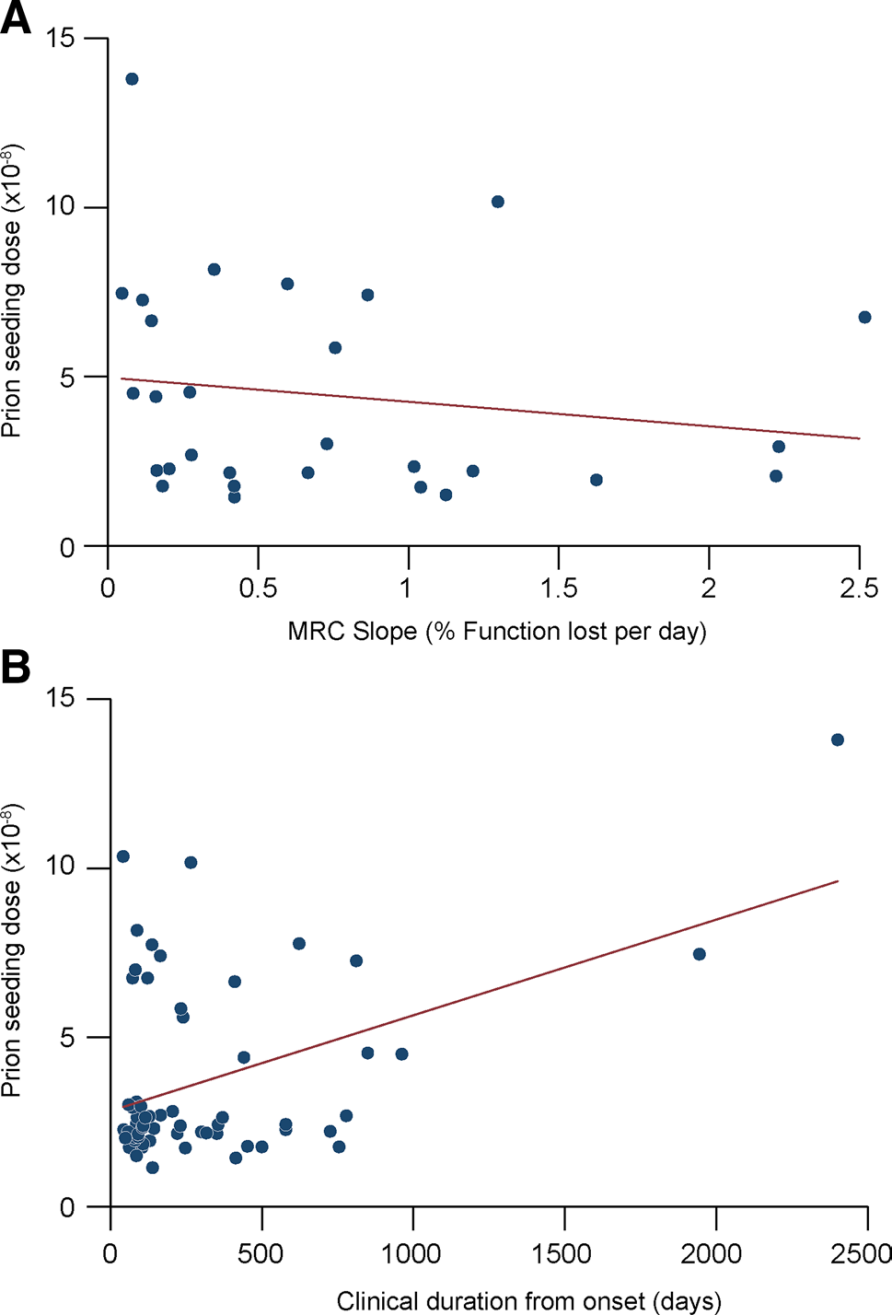
Correlation of CSF prion seeding doses with functional decline and duration of illness. Correlation of the MRC Scale slope (percentage decline in function per day) with prion seeding dose by RT-QuIC (**A**), and Correlation of the clinical duration from symptom onset with prion seeding dose by RT-QuIC (**B**). No correlation was seen with rate of functional decline (**A**) but a positive correlation was found (*r* = 0.44,* p* = 0.0004) with total duration of illness. However the latter positive correlation is dependent on 2 extreme outliers with long durations of illness.

The rate of decline in individual MRC Scale scores from RT-QuIC positive CSF samples were calculated according to the mixed effects model described in Mead et al*.*^28^. Out of the 70 sCJD CSF samples that were scored positive, 61 had at least one MRC score recorded, but only 29 of 61 had sufficient data compiled to generate individual rates of MRC Scale score decline (percentage decline per day). In these 29 individuals, no significant correlation was found between prion seeding dose and functional decline (r = − 0.16, p = 0.41) (Fig. 5A). This effect remained when patients were separated into their respective *PRNP* codon 129 genotypes (MM (n = 9), r = 0.13, p = 0.73; MV (n = 15), r = − 0.34, p = 0.21; VV (n = 5), r = − 0.88, p = 0.05).

When the CSF prion seeding dose was compared against the total duration of disease available for all 61 patients instead of rate of MRC Scale score decline, a positive correlation was found (*r* = 0.44,* p* = *0.0004*), i.e. the longer the duration of disease, the higher the seeding dose (Fig. 5B). The correlation coefficients for the MM (n = 28), MV (n = 19) and VV (n = 11) groups are 0.08 (p = 0.68), 0.56 (p = 0.02) and − 0.006 (p = 0.98) respectively; the positive correlation in the MV group is largely accounted for by 2 outliers with extraordinarily long durations of disease (1944 days and 2401 days), over double that of any other patient.

## Discussion

This study shows, for the first time in a sizeable sample set, that RT-QuIC reactions using BV rPrP as the conversion substrate can be successfully seeded by human prions in CSF, albeit autopsy-derived. Whilst the RT-QuIC had previously been adapted to the 384-well format, it was confined to sheep scrapie BH seeds^30^; here, we show that this is similarly applicable to CSF containing human prions.

Interest in exploring the utility of BV rPrP in CSF RT-QuIC here followed a series of benchmark experiments carried out by Orrú et al. 2015 in which BV rPrP appeared to show potential as a 'universal' acceptor in a wide range (but small numbers of n = 2–4 per species) of mammalian prion species^15^. Of particular interest were positive results from human BH samples of so-called GSS-causing mutations (P102L, F198S, A117V, H187R) with low molecular weight fragments on western blot, and D178N-FFI which had previously yielded negative results in reactions using hamster rPrP even with enhanced IQ-CSF conditions^19^. Furthermore, the failure of IQ-CSF RT-QuIC iterations (with either full length or truncated hamster rPrP) to uniformly replicate their enhanced sensitivity in non-fCJD *PRNP* mutation samples emphasises this unmet clinical need for a universal acceptor^18,20,21,31^.

In the largest series of sCJD PM CSF samples in the literature tested with BV rPrP RT-QuIC to date, both the sensitivity and specificity of 88.6% (95% CI 79.5–94.7%) and 91.2% (95% CI 80.7–97.1%) respectively in our study are below that reported in a number of recent large non-BV rPrPstudies (sensitivities 91–97.2%, specificities 98–100%) in which the reactions are seeded by ante mortem CSF^14,18,20,21,31^. In our study, BV rPrP also appear to be successfully converted by other prion disease subtypes such as vCJD and a range of IPDs. While E200K, P102L and D178N CSF have been shown to seed RT-QuIC reactions in other studies using different rPrP including full length Hu rPrP, full length hamster rPrP, truncated Syrian hamster rPrP (90–231) and hamster-sheep chimeric rPrP^20–22,31,32^, our study here showed for first time that CSF from cases of 6-OPRI IPD can seed RT-QuIC reactions.

The lower sensitivity and specificity of CSF BV rPrP RT-QuIC in sCJD, and particularly its even lower sensitivity across IPD subtypes appear to be at variance with the findings in Orrù et al.^15^, and suggest that perhaps BV rPrP may not be a completely “universal acceptor” of prion strains. Moreover, we are aware of a selection of 19 CSF samples (from a small collection of 22 sCJD and 4 fCJD (E200K) ante mortem CSF) tested under BV rPrP RT-QuIC conditions similar to Orrù et al.^15^ which returned an even lower sensitivity relative to our study of 52.6%^33^. Possible explanations for our discrepancies may lie in the differential sample matrix and RT-QuIC conditions, and the presence of ‘conversion’ barriers, or a combination thereof.

Firstly, the use of PM CSF in this pilot study is relatively unconventional as well-established CSF RT-QuIC protocols in use are honed chiefly for ante mortem CSF which has a different fluid matrix to PM CSF, and PM CSF is expected to have lower seeding activity compared to BH previously tested with BV rPrP RT-QuIC. However, given the large volumes of PM CSF that can be collected relative to ante mortem CSF, we judged it to be an appropriate biofluid medium to conduct an exploratory study in a reasonably large sample set. Key CSF composition factors identified to have profound effects on RT-QuIC reaction kinetics include red cell count (haemoglobin concentration), white cell count and protein level. The haem concentration in CSF samples with red cell counts exceeding 1250 × 10^6^/L may quench the fluorescent signal sufficiently to return false negative results^34^; on the other hand, protein levels greater than 1.0 g/L and raised white cell count beyond 10 × 10^6^/L are liable to cause false positive misinterpretations due to high and fluctuating baseline rfu measurements^35^. It is recognised that significant physicochemical changes take place in death, even in closed compartment fluids like CSF^36^; broadly speaking, leakage of impurities into PM CSF which follows cessation of active transport mechanisms that maintain the blood–brain barrier such as cell debris and proteins can inhibit amyloid seeding assays^37–39^. More specifically, CSF total protein level is known to increase significantly by about 20 times (mean 8.37 g/L) in agonal death durations greater than 6 h such as that observed in prion disease^40^, and perhaps further increased with longer death to autopsy intervals^41^. As for PM CSF white cell count, it increases progressively with post mortem interval, and autopsy studies indicate that CSF cell count would have exceeded 10 × 10^6^/L by 10 h post mortem^42^.

Cell counts and routine biochemistry were not performed on our PM CSF sample set in clinical laboratories for biohazard concerns, although visually pink or red samples indicating significant red cell contamination were discarded from analyses. In spite of this, it remains likely that the PM CSF samples used here (with mean PM intervals of 5.6 days) contain total protein levels and white cell counts (or its debris) greatly in excess of 1.0 g/L and 10 × 10^6^/L respectively. Indeed, all 5 of our positive control PM CSF samples were scored as such due to high and fluctuating baseline. These 5 reaction kinetics and curves were distinct from conventional positive results, and arguably should have been considered ‘negative’ from a *qualitative* perspective. These anonymised control samples were obtained from a different institution where only the ages of death and gender were provided; these patients died of non-neurological causes (and thus presumably non-prion), some of which involve trauma, but the precise cause of death for each individual was not provided. As a result, we did not have the resources to age- and gender-match all cases for this exploratory study.

Aside from the different seed matrices between Orrù et al.^15^ and our study, other differences are summarised in Table 3. Overall, it is difficult to discern if a singular factor within these could account for the discrepancy, notwithstanding the expected lower seed concentration in CSF compared to BH. However limited experience with ante mortem CSF BV rPrP RT-QuIC to date does suggest that reaction efficiency for BV rPrP RT-QuIC is not as readily replicated between seed matrices compared to IQ-CSF using truncated hamster rPrP^19,20^. One may be tempted to raise the reaction temperature but BV rPrP RT-QuIC has tended to spontaneously fibrillise between the 60–70th hour even at a 42 °C^15^.

**Table 3:** Comparison of RT-QuIC key conditions used in this study against those of Orrù et al*.*^15^ and Vallabh et al*.*^33^.

RT-QuIC conditions	Our study	Orrù et al*.*^15^	Vallabh et al*.*^33^
**Reaction mix components/well**			
Phosphate buffer (pH 7.4)	10 mM	10 mM	10 mM
NaCl	300 mM	300 mM	300 mM
EDTA	10 µM	1 mM	1 mM
ThT	10 µM	10 µM	10 µM
SDS	None	0.001%	0.001%
rPrP	0.1 mg/mL	0.1 mg/mL	0.1 mg/mL
H_2_O	As required to make up respective well volume		
Seed type	PM CSF	BH	AM CSF
See volume per well	7.5 µL	2 µL	20 µL
Total volume/well	50 µL	100 µL	100 µL
Microplate format	384-well	96-well	96-well
BV rPrP construct	Cleaved *his*-tagged	Non *his*-tagged	Non *his*-tagged
Reaction temperature	50 °C	42 °C	42 °C

*NaCl* sodium chloride, *EDTA* ethylenediaminetetraacetic acid, *ThT* thioflavin T, *SDS* sodium dodecyl sulphate, *rPrP* recombinant prion protein, *PM CSF* post mortem cerebrospinal fluid, *AM CSF* ante mortem cerebrospinal fluid, *BH* brain homogenate, *his-tagged* histidine-tagged.

One other key consideration is the possibility that there may still exist “conversion” barrier(s) with BV rPrP RT-QuIC if differential seeding activity and matricial compositional factors are insufficient to explain the result discrepancy. The collated literature does suggest that CSF from *PRNP* mutations with a fCJD phenotype, in particular the E200K mutation, can easily seed RT-QuIC reactions, but less so in others which produce the GSS or FFI phenotypes^20,21,31^. What this is down to is not entirely clear but this disparity seemed to be resolved by moving towards rPrP sequence homology where RT-QuIC conversion rates between different IPDs were shown to be comparable by using full length Hu rPrP^22^; Sano et al. in 2013 achieved positive RT-QuIC reactions from E200K (18/22), P102L (18/20) and D178N-FFI (10/12) CSF samples by using full length Hu rPrP^22^. However it ought to be pointed out that the reaction solution composition, temperature and microplate reader is significantly different to the RT-QuIC protocols employed by us and Orrù et al.^15^. Mouse modelling studies with GSS-related P102L and A117V prions where prions derived from the respective mutant protein can only be transmitted to transgenic mice expressing the corresponding mutant human PrP, and not to wild type mice or mice expressing wild type human PrP^23,24^, may give this further traction. It is thus possible that prions from certain IPD mutations may require mutant human rPrP to allow conversion in RT-QuIC reactions.

The RT-QuIC assay is largely perceived as a “*reporting*” assay in which the outcome is binary, though attempts have been made at probing RT-QuIC kinetics and products to elucidate the prion seeding dose in test samples. RT-QuIC end-point dilution remains the most accurate way of measuring seeding-competent PrP conformers in a test sample in place of animal bioassay^38^, but the quantitative method proposed by Shi et al. (similar to that employed here) based on correlating dose against reaction lag times on a calibrated curve, appears to promise efficiency in terms of time and spatial requirements^29^. CSF is routinely only drawn once for diagnostic purposes, and regular CSF sampling at intervals during the illness is hard to justify both clinically and ethically. On the expectation that PM CSF contains the “final and maximal" CSF prion seeding dose, we explored if the extrapolated dose scorrelates with the rates of functional decline in these patients, measured by the percentage decline per day of the validated MRC Scale score obtained in life^27,28^. In this study, no clear correlation was found between seeding dose and rate of MRC Scale score (functional) decline. A moderate positive correlation was counterintuitively observed between prion seeding dose and total illness duration across all patients; however, once segregated into codon 129 genotypes this relationship remained statistically significant only for patients with the MV genotype, and could be accounted for by a mere 2 outliers with extremely long durations of illness. This is perhaps unsurprising as it is not precisely known whether a single or multiple species of permissible conformational strains of prions are present within any single biofluid sample and which ones are capable of seeding RT-QuIC reactions^43^. Moreover, the two-phase kinetic model of prion propagation distinguishes infectious prions and neurotoxic PrP species whose production is uncoupled^44,45^. In other words, the abundance of the toxic species mediating functional decline may not be reflected in RT-QuIC seeding competency, and vice versa. Also, rate of decline data was only available from about a third of sCJD patients in the sample set, and as such the numbers may be too small to draw any firm conclusions at the present time.

Our pilot study demonstrated that BV rPrP RT-QuIC can be successfully seeded by PM CSF from a relatively large and diverse sample set derived from prion disease patients, and that it can be adapted to the 384-well format to increase throughput and conserve limited CSF volumes. Significant difference remains between its efficiency for sCJD and IPD groups, and as such is at variance with its previously shown “universality” with prion species^15^. This however does not necessarily invalidate BV rPrP’s potential as a “universal acceptor” of prions as the disparities may merely reflect differences in the matrices of test samples (and hence their seeding doses) and/or in the RT-QuIC conditions used. Nevertheless, we believe our results justifies further optimisation of this assay for wider diagnostic and prognostic use.

## Methods

### Research governance, clinical data, post mortem examination and sample preparation

CSF and brain tissue used in this study were obtained at post mortem from patients who were suspected to have prion disease while alive, and subsequently archived at the Medical Research Council Prion Unit at UCL. The study was performed with approval from the London—Queen Square Research Ethics Committee (Reference: 03/N022). Fully informed consent was obtained, and samples procured and used in accordance with the approved study and relevant national legislation and guidelines.

The majority of these patients were enrolled either into the National Prion Monitoring Cohort (NPMC) or the MRC PRION-1 Trial^46^ the autopsies were largely performed at the National Hospital for Neurology & Neurosurgery on Queen Square or less frequently in local hospitals, both in rooms designated for high-risk autopsies. Full research consent was obtained for all samples used in this study; Scotland A Research Ethics Committee (NPMC) or the Eastern Research Ethics Committee (PRION-1). Prion disease patients enrolled into the NPMC and PRION-1 Trial had regular clinical assessments and their functional status recorded as the Medical Research Council Prion Disease Rating Scale (MRC Scale x/20) over time^27,46^.

At each autopsy, CSF was aspirated by pipette from the pontine cistern by access through the skull base; this is achieved sequentially by skull removal, separation of dura, lifting of frontal and temporal lobes, and separations of the optic chiasm from the skull base and pituitary gland from pituitary stalk respectively. All CSF samples were stored at − 80 °C prior to analysis. CSF from neuropathologically confirmed prion cases were obtained from MRC Prion Unit at UCL. Fully anonymised non-neurological control post-mortem (PM) CSF samples were obtained from Dr Esiri at the Oxford Brain Bank.

Brain homogenates (BH; 10% w/v) were prepared as previously described^6^ and stored at − 80 °C. Aliquots of 10% w/v BH were thawed at room temperature freshly for each RT-QuIC assay. The BHs were diluted serially in artificial CSF (150 mM NaCl, 2 Mm CaCl_2_, 1.2 mM MgCl_2_, 1.5 mM K_2_HPO_4_ and 10 mM glucose, pH 7.3) in the quantitative RT-QuIC assay.

### Expression and purification of full length human (Hu rPrP) and bank vole (BV rPrP) recombinant PrP

Hu rPrP (aa residues 23–231; with methionine at codon 129; accession M13899) and BV rPrP (aa residues 23–231; with methionine at codon 129; accession AF367624) were prepared according to Jackson et al*.* 1999 with minor modifications^47^. Briefly, the DNA sequences encoding either rPrP in pTrcHisB were transformed into BL21 (DE3). BL21 cultures were grown in LB medium in the presence of 100 µg/ml Ampicillin. Expression of either rPrP was then induced using 1 mM IPTG, and purified from inclusion bodies under denaturing conditions using Nickle superflow resin with an AKTA Pure (GE Healthcare Life Sciences). Subsequently, the rPrP was eluted from the column using an imidazole gradient, and the eluted material was extensively dialysed against 20 mM Bis Tris pH 6.5. The rPrP Histidine tag was cleaved with the addition of 2.5 mM CaCl_2_ and 50U Thrombin (VWR). Following this, the rPrP was run through a second NiNTA on the AKTA Pure to remove the Histidine tag, and dialysed against 10 mM Bis Tris pH 6.5. The concentration of rPrP was determined by absorption at 280 nm with molar extinction coefficients of 61,880 M^−1^ cm^−1^ and 56,667 M^−1^ cm^−1^ for BV rPrP and Hu rPrP respectively. Recombinant PrP concentration was adjusted to 0.2–0.5 mg/mL by the addition of buffer. Aliquots were stored at − 80 °C until use. Prior to usage, aliquot(s) of rPrP was dialysed into 20 mM Sodium phosphate buffer (pH 5.8), filtered (0.22 μm syringe).

### RT-QuIC analysis of CSF and BH

The RT-QuIC reaction buffer was composed of 10 mM phosphate buffer (pH 7.4), 300 mM NaCl, 0.1 mg/mL rPrP, 10 μM Thioflavin T (ThT), and 10 μM ethylenediaminetetraacetic acid (EDTA). Reactions were prepared in either 96-well or 384-well optical clear-bottomed plates (Nalgene Nunc International 265301; 242764). For 96-well plates, each well contained either 98 μL of reaction buffer seeded by 2 μL of BH or 85 μL of reaction buffer seeded by 15 μL CSF, bringing the total volume in each well up to 100 μL. The proportion of the components, and hence its concentrations were maintained when applied to different volumes in 384-well plate experiments e.g. 42.5 μL reaction buffer seeded by 7.5 μL CSF (total 50 μL). Each CSF sample was run in quadruplicate, allowing for 5 negative controls (PM non-prion CSF) and 5 positive controls (definite sCJD CSFs).

Thereafter, the plates were sealed and incubated in a BMG OPTIMA FLUOstar plate reader at either 45 °C or 50 °C for 90–120 h with cycles of intermittent shaking. Each cycle consisted of 60 s of double orbital shaking at 700 rpm followed by 60 s rest, with ThT fluorescence measurements (450 nm excitation; 480 nm emission) taken every 2 min. The plate reader measures ThT fluorescence in relative fluorescence units (rfu) with a maximum rfu at 260,000. A reaction was considered positive if the average rfu in 2 or more wells exceeded a threshold rfu defined as the average rfu from 5 negative controls plus 5 standard deviations, and within 90 h of start time.

### Statistical analysis

The sensitivity and specificity were calculated for sCJD and IPD groups, and statistical significance between groups was determined by Fisher's Exact Test (Stata, v15.1). Mixed effect linear modelling of MRC Scale scores was undertaken with Stata, v15.1.
